# PD-1 checkpoint inhibition enhances the antilymphoma activity of CD19-CAR-iNKT cells that retain their ability to prevent alloreactivity

**DOI:** 10.1136/jitc-2023-007829

**Published:** 2024-01-31

**Authors:** Emmanuelle Moraes Ribeiro, Kathy-Ann Secker, Ana-Maria Nitulescu, Rebekka Schairer, Hildegard Keppeler, Anton Wesle, Hannes Schmid, Anita Schmitt, Brigitte Neuber, Daniela Chmiest, Silvia Podavini, Melanie Märklin, Boris Klimovich, Michael Schmitt, Fulya Korkmaz, Claudia Lengerke, Corina Schneidawind, Dominik Schneidawind

**Affiliations:** 1 Department of Hematology, Oncology, Clinical Immunology and Rheumatology, University Hospital Tübingen, Tübingen, Germany; 2 Department of Oncology, Hematology and Rheumatology, University Hospital Heidelberg, Heidelberg, Germany; 3 Department of Immunobiology, University of Lausanne, Lausanne, Switzerland; 4 Clinical Collaboration Unit Translational Immunology, German Cancer Consortium (DKTK), Department of Internal Medicine, University Hospital Tübingen, Tübingen, Germany; 5 Cluster of Excellence iFIT (EXC 2180) "Image-Guided and Functionally Instructed Tumor Therapies", University of Tübingen, Tübingen, Germany; 6 Department of Medical Oncology and Hematology, University Hospital Zurich, Zurich, Switzerland

**Keywords:** Immunotherapy, Transplantation Immunology, Natural Killer T-Cells, Receptors, Chimeric Antigen, Immune Checkpoint Inhibitors

## Abstract

**Background:**

Relapse and graft-versus-host disease (GVHD) are the main causes of death after allogeneic hematopoietic cell transplantation (HCT). Preclinical murine models and clinical data suggest that invariant natural killer T (iNKT) cells prevent acute and chronic GVHD. In addition, iNKT cells are crucial for efficient immune responses against malignancies and contribute to reduced relapse rates after transplantation. Chimeric antigen receptors (CAR) redirect effector cells to cell surface antigens and enhance killing of target cells. With this study, we aimed to combine enhanced cytotoxicity of CD19-CAR-iNKT cells against lymphoma cells with their tolerogenic properties.

**Methods:**

iNKT cells were isolated from peripheral blood mononuclear cells and transduced with an anti-CD19-CAR retrovirus. After in vitro expansion, the functionality of CD19-CAR-iNKT cells was assessed by flow cytometry, image stream analysis and multiplex analysis in single-stimulation or repeated-stimulation assays. Moreover, the immunoregulatory properties of CD19-CAR-iNKT cells were analyzed in apoptosis assays and in mixed lymphocyte reactions. The effect of checkpoint inhibition through nivolumab was analyzed in these settings.

**Results:**

In this study, we could show that the cytotoxicity of CD19-CAR-iNKT cells was mediated either through engagement of their CAR or their invariant T-cell receptor, which may circumvent loss of response through antigen escape. However, encounter of CD19-CAR-iNKT cells with their target induced a phenotype of exhaustion. Consequently, checkpoint inhibition increased cytokine release, cytotoxicity and survival of CD19-CAR-iNKT cells. Additionally, they showed robust suppression of alloreactive immune responses.

**Conclusion:**

In this work, we demonstrate that CAR-iNKT cells are a powerful cytotherapeutic option to prevent or treat relapse while potentially reducing the risk of GVHD after allogeneic HCT.

**WHAT IS ALREADY KNOWN ON THIS TOPIC**

- CAR-T-cell therapy has revolutionized the treatment of hematological cancers. Nevertheless, this novel immunotherapeutic approach has still some considerable limitations such as production failure, side effects, relapse rates and limited use in distinct clinical situations such as allogeneic hematopoietic cell transplantation (HCT). Here, the use of donor T cells, the risk of graft-versus-host disease (GVHD) and relapse through antigen escape constitute particular problems. Invariant natural killer T (iNKT) cells are a small T-cell subset that induces immune tolerance and prevents GVHD while containing cytotoxic features. Thus, iNKT cells might have some benefits as CAR effector cell population.

**WHAT THIS STUDY ADDS**

- This study represents a comprehensive analysis about CD19-CAR-iNKT cells for the treatment of lymphoid malignancies after allogeneic HCT. We highlight the advantages of this particular effector cell population based on its immunoregulatory properties. CAR-iNKT cells may be used after allogenic HCT without the risk of GVHD. Even checkpoint inhibition could not override this feature and independent killing pathways could circumvent relapse based on antigen escape.

**HOW THIS STUDY MIGHT AFFECT RESEARCH, PRACTICE OR POLICY**

- This study provides evidence that T cells are not the optimal CAR effector cell population for particular clinical situations such as allogeneic HCT. We suggest that iNKT cells have distinct advantages over conventional T cells. A more differentiated approach regarding the optimal selection of effector cells in distinct clinical situations is required.

## Introduction

Relapse and graft-versus-host disease (GVHD) are the main causes of death after allogeneic hematopoietic cell transplantation (HCT). Graft-versus-leukemia (GVL) effects and GVHD are both mediated through donor T cells. Efforts to prevent GVHD jeopardize beneficial GVL effects and this tradeoff does not necessarily translate into improved overall survival.

Invariant natural killer T (iNKT) cells are a small T-cell subset that plays a significant role in malignancies, infectious diseases, autoimmunity, graft rejection and alloimmunity.^1–3^ They are characterized by the expression of an invariant T-cell receptor.^4^ In contrast to conventional T cells, iNKT cells recognize glycolipids presented by the MHC-I-like molecule CD1d. On activation, they release large amounts of cytokines and effector molecules such as IL-4, IFN-γ, TNF-α, perforin and granzymes.^5–7^

We have previously shown that human iNKT cells promote immunotolerance after allogeneic HCT by inducing preferential apoptosis of conventional dendritic cells (DCs) leading to a bias in favor of plasmacytoid DCs. This results in a reduced activation and proliferation of alloreactive T cells.^8^ Furthermore, the ability of iNKT cells to prevent GVHD has been demonstrated in several murine models and clinical studies.^9–12^ On adoptive transfer, iNKT cells inhibit donor T-cell expansion, induce a Th2 bias and expand donor FoxP3+CD4+ regulatory T cells through myeloid-derived suppressor cells.^13^ In these models, we also observed that donor iNKT cells are capable of reducing lymphoma burden when transferred with a T-cell depleted graft.^14^ This demonstrates relevant graft-versus-tumor effects of tolerogenic iNKT cell.

iNKT cells exert cytotoxicity against malignant cells through effector molecules such as perforin and granzymes. Moreover, they cross-prime other lymphocytes to mediate cytotoxicity.^15 16^ However, chronic stimulation of iNKT cells results in exhaustion that can be overcome by blocking checkpoint molecules such as PD-1.^17^ Nelson *et al* demonstrated recently that iNKT cells showed superior tumor control with PD-1 checkpoint inhibition in a murine model of pancreatic ductal adenocarcinoma.^18^

Specificity and cytotoxicity of effector cells can be further increased through the introduction of a chimeric antigen receptor (CAR). CAR-T-cell therapy has shown promising results for the treatment of several lymphoid malignancies.^19 20^ It is also an attractive approach to enhance GVL effects to prevent and treat relapse after allogeneic HCT.^21 22^ However, the use of T cells modified to express a CAR (CAR-T cells) may also increase the risk for GVHD in this setting, especially in combination with checkpoint inhibition.^23–25^ This is crucial since transduction efficiency is highly variable and non-transduced T cells may constitute the major fraction of a CAR-T-cell product. Also, with the increasing use of haploidentical donors, the major histocompatibility barrier constitutes a major threat to induce severe GVHD with a given dose.

Thus, CAR-iNKT cells would represent an elegant way to prevent or treat relapse after allogeneic HCT without causing GVHD. In contrast, they might even lower the incidence of GVHD after allogeneic HCT. Reduced relapse rates and improved non-relapse mortality would result in improved overall survival. Several studies have evaluated the feasibility of CAR-iNKT cells against various malignancies such as B-cell lymphoma, neuroblastoma and myeloma.^26–29^ In particular, CD19-CAR-iNKT cells showed robust in vitro and in vivo activity against brain lymphomas resulting in improved survival of mice.^26^ Also, CAR-iNKT cells cross-prime effector T cells indicating that they retain their immunoregulatory properties despite transduction.^30^ Currently, several clinical trials are underway investigating CD19-CAR-iNKT cells to treat lymphoma in humans (eg, NCT04814004, NCT00840853): Allogeneic CAR-iNKT cells are well tolerated and induce objective responses.^31^

In the present work, we performed a comprehensive analysis of iNKT cells transduced with a third-generation CAR as an off-the-shelf immunotherapeutic strategy against various CD19+ malignancies. We studied whether checkpoint inhibition would have a beneficial effect on their cytotoxic functionality. Notably, their tolerogenic properties were analyzed in presence of nivolumab, which usually enhances alloreactive immune responses of T cells stimulated by DCs.

## Materials and methods

### Research subjects

Human buffy coats from healthy volunteers were obtained from the Center of Clinical Transfusion Medicine Tuebingen. Leukemia blasts were isolated from patients prior to treatment after written informed consent has been obtained. HLA typing was performed by the Center of Clinical Transfusion Medicine Tuebingen or the HLA laboratory of the Department of Medicine II of the University Hospital Tuebingen.

### CD19-CAR-iNKT-cell generation

The generation of CD19-CAR-iNKT cells was performed with a CD19-specific third-generation (CD19.CAR-CD28/CD137/CD3ζ) retroviral CAR construct. The retroviral supernatants were produced as described previously.^32^ iNKT cells were isolated upfront from peripheral blood mononuclear cells (PBMCs) using anti-iNKT MicroBeads (Miltenyi Biotech, Bergisch Gladbach, Germany) according to the manufacturer’s instructions. The cells of the flow-through were loaded with α-galactosylceramide (α-GalCer, Sigma-Aldrich, St. Louis, USA) for 4 hours, irradiated with 30 Gy and used as feeder cells. Isolated iNKT cells were activated with α-GalCer-loaded autologous feeder cells (1:2 iNKT cell to feeder cells ratio), 200 I.U./mL recombinant human interleukin 2 (rhIL-2, Novartis, Basel, Switzerland) and 200 ng/mL α-GalCer in CAR medium containing 45% EHAA (Clicks) medium (Irvine Scientific, Santa Ana, USA), 45% RPMI 1640 (ThermoFisher Scientific, Waltham, USA), 2 mM L-glutamine (Lonza, Basel, Switzerland) and 10% FBS (fetal bovine serum, Sigma-Aldrich). The remaining α-GalCer-loaded autologous feeder cells were cryopreserved for later (CAR)-iNKT-cell expansion. After 72 hours, activated iNKT cells were transduced with viral supernatant, using retronectin (Takara Bio Europe, Saint-Germain-en-Laye, France) as transduction enhancer. On day 6, transduced cells were harvested, restimulated with α-GalCer-loaded autologous feeder cells, 100 I.U./mL rhIL-2 and 100 ng/mL α-GalCer. Restimulation was repeated after 7 days. Transduction efficiency and cell expansion was determined on day 20.

### Flow cytometry

PBS57-loaded and unloaded human CD1d tetramers were obtained from the National Institutes of Health Tetramer Core Facility (Atlanta, USA). The following antibodies were purchased from BD Biosciences (Franklin Lakes, USA) or BioLegend (San Diego, USA): anti-CD19 (HIB19), anti-CD25 (BC96), anti-CD28 (CD28.2), anti-CD3 (HIT3a, OKT3), anti-CD4 (RPA-T4, OKT4), anti-CD44 (IM7), anti-CD45RA (HI100), anti-CD69 (FN50), anti-CD8 (HIT8a), anti-CTLA-4 (BNI3), anti-IgG1 (mouse, MOPC-21), anti-IgG2b (mouse, MPC-11), anti-IgG1 (mouse, MOPC-21), anti-IgG2b (mouse, MOPC-173), anti-KLRG1 (14C2A07), anti-LAG-3 (11C3C65), anti-PD-1 (EH12.2H7), anti-PD-L1 (MIH2), anti-PD-L2 (24F.10C12), anti-TIGIT (A15153G), anti-TIM-3 (F38-2E2). A goat F(ab’)2 anti-human IgG (H+L)-F(ab’)2-fragment (polyclonal, Jackson Immunoresearch, Ely, UK) and the CD19-CAR detection reagent (Miltenyi Biotech) were used to determine the percentage of CAR-expressing cells. Fluorescence minus one controls were used for proper gating. To stain dead cells, eBioscience Fixable Viability Dyes eFluor 506, 780 (ThermoFisher Scientific) and 7-AAD (7-aminoactinomycin, BD Biosciences) were used. Data were acquired on a BD LSRFortessa cell analyzer (BD Biosciences) and analyses were performed with FlowJo V.10.2 (Tree Star, Ashland, USA).

### Cytotoxicity assays

Cytotoxicity was assessed with an annexin V-FITC staining kit (Miltenyi Biotech) or by ImageStream analysis. The viability dye was chosen based on the panel necessary for flow cytometric analyses. Except for DCs, all target cells were stained with CellTrace Violet (CTV, Thermo Fisher Scientific) prior to the cytotoxicity assays. For that, cells were centrifuged (5 min, RT, 450xg) and the supernatant was completely removed. Cells were resuspended in 1 mL DPBS and stained with 500 nM CTV for 20 min (37°C, protected from light). Afterwards, the reaction was stopped with medium containing 10% FBS and the cells were washed once. CD19-CAR-iNKT cells or untransduced iNKT cells were incubated for 4–24 hours with CTV+ target cells (SEM, Raji, Jurkat, K562 cells or patient samples) or DCs at determined effector to target ratios (E:T). After coincubation, the cells were stained and analyzed by flow cytometry. Where stated, 10 µg/mL nivolumab (Novartis) or its IgG4 isotype control (BioLegend), 100 ng/mL α-GalCer (Abcam, Cambridge, UK) or dimethyl sulfoxide (DMSO, Sigma-Aldrich) were added to the assays. The results are expressed as percentage of cytotoxic activity using the formula: cytotoxic activity (%)=(1−(% annexin V^neg^viability dye^neg^ target cells incubated with CD19-CAR-iNKT cells/% annexin V^neg^viability dye^neg^ target cells incubated alone))×100.

### Carboxyfluorescein succinimidyl ester dilution

The proliferation of CD19-CAR-iNKT cells and T cells was determined by carboxyfluorescein succinimidyl ester (CFSE) dilution. First, CD19-CAR-iNKT cells or MACS-isolated T cells were resuspended in phosphate-buffered saline (PBS, Gibco) and stained with CellTrace CFSE cell proliferation kit (BioLegend) for 5 min at room temperature. Immediately after staining, cells were washed with pure FBS and then two times in PBS supplemented with 5% FBS and finally resuspended in CAR medium. CFSE-labeled cells were tested in a mixed lymphocyte reaction (MLR) and against modified Raji cells. Where stated, 10 µg/mL nivolumab (Novartis) or its IgG4 isotype control (BioLegend) was added. Results are expressed as a percentage of proliferating cells or as mean fluorescence intensity.

### Mixed lymphocyte reactions

To generate monocyte-derived DCs (mo-DCs), plastic-adherent monocytes isolated from PBMCs were cultured for 6 days in RPMI 1640 GlutaMAX Medium (ThermoFisher Scientific), 10% FBS (Sigma-Aldrich), 100 I.U./mL penicillin-streptomycin (Lonza), 11.4 µM 2-mercaptoethanol (Roth, Karlsruhe, Germany), 0.1 mM non-essential amino acids (Gibco, Grand Island, USA) and 1 mM sodium pyruvate (Gibco) supplemented with 50 ng/mL IL-4 and 100 ng/mL GM-CSF (Miltenyi Biotec) every other day. Major mismatched mo-DCs (stimulators) were plated together with allogeneic T cells (responders) at a 1:1 ratio and 5-fold third-party donor CD19-CAR-iNKT cells or untransduced iNKT cells. Where stated, 10 µg/mL nivolumab (Novartis) or its IgG4 isotype control (BioLegend) were added to the assays. Cells were analyzed by flow cytometry for activation markers (CD69 or CD25) and proliferation (CFSE dilution) on day 1, 3 and 7, respectively.

### Multiplex cytokine analysis

Cell culture supernatants from cytotoxic assays were collected after their respective incubation time. Cytokine release was measured by a LEGENDplex human CD8/NK cells panel (BioLegend), according to the manufacturer’s instructions. Data were acquired using the BD FACSLyric clinical cell analyzer with autosampler (BD Biosciences) and analyzed with the LEGENDplex Data Analysis Software Suite.

### Statistical analysis

Student’s t-test or analysis of variance was used for statistical analyses and p<0.05 was considered statistically signiﬁcant. Data were analyzed with Prism V.9 (GraphPad Software, La Jolla, USA). All experiments were performed with at least three different iNKT-cell donors, in duplicates or triplicates and repeated at least twice independently.

## Results

### CD19-directed CAR-iNKT cells show robust cytotoxicity against CD19+/CD1d+ target cells

**Figure 1:**
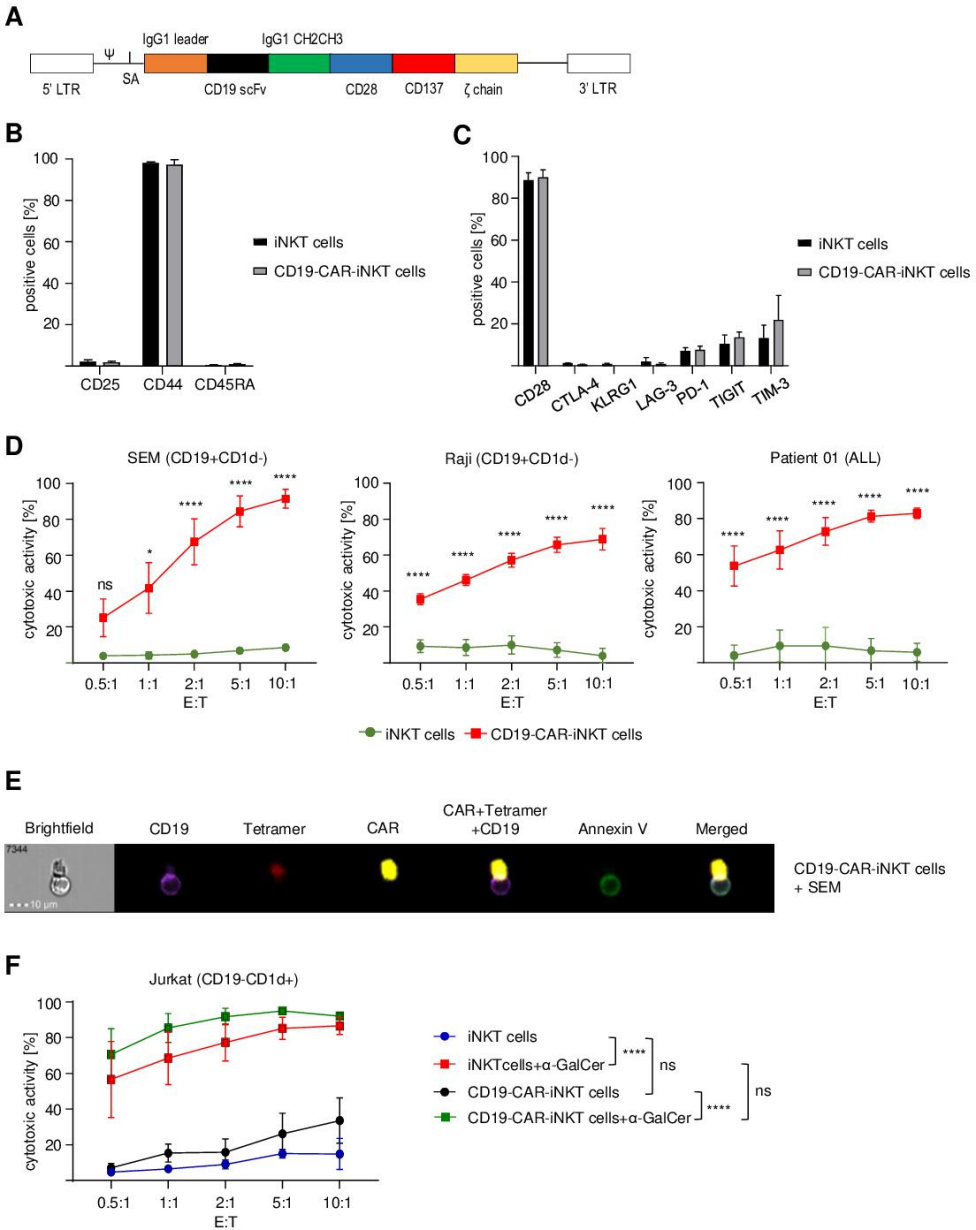
CD19-directed CAR-iNKT cells induce cell death of CD19+ cell lines and primary patient cells. (A) Schematic representation of the anti-CD19-CAR-CD28/CD137ζ construct. (B) Flow cytometric analysis of the phenotype of untransduced iNKT and CD19-CAR-iNKT cells on day 20 (n=3). (C) Exhaustion profile of CD19-CAR-iNKT cells on day 20 (n=3). Data were obtained after gating on live (viability dye−, VD−) lymphocytes (FSC-A vs SSC-A) and singlets (SSC-A vs SSC-W). (D) Cytotoxic activity of CD19-CAR-iNKT cells against an ALL cell line (SEM), a Burkitt lymphoma cell line (Raji) and primary blasts isolated from an ALL patient after 4 hours of coculture (n=3). (E) Representative ImageStream analysis showing apoptosis induction (green) of an ALL cell line (SEM, purple) after direct contact with a CD19-CAR-iNKT cell (red and yellow) after 4 hours of coincubation. (F) CD1d-dependent cytotoxicity of CD19-CAR-iNKT cells against Jurkat cells (CD19−CD1d+) after 4 hours (n=3). Data were obtained after gating on all cells (FSC-A vs SSC-A), singlets (SSC-A vs SSC-W) and annexin V−VD−CTV+ cells. All graphs show pooled data of at least three independent iNKT-cell donors tested in at least two independent experiments. The cytotoxicity of the effector cells was assessed in three technical replicates. Error bars indicate SE of the mean (SEM), ns p>0.05, *p≤0.05, ****p≤0.0001. Two-way ANOVA was used for statistical analysis. ANOVA, analysis of variance; iNKT, invariant natural killer T.

Despite being rare in peripheral blood, iNKT cells can be easily expanded ex vivo to achieve cell numbers, which are necessary for clinical applications. For the generation of CD19-CAR-iNKT cells, the original protocol for T cells had to be adapted considering several unique properties of iNKT cells.^32^ The protocol, which was established for this work, consisted of upfront iNKT-cell isolation and activation using α-GalCer-loaded autologous feeder cells, IL-2 and α-GalCer (online supplemental data 1A). After transduction with a retroviral SFG.CD19-CAR construct (figure 1A), iNKT cells were expanded during two stimulation rounds (weekly) with feeder cells, IL-2 and α-GalCer. This protocol allowed a significant expansion of iNKT cells (47.5-fold±9.3 for CD19-CAR-iNKT cells and 95.0-fold±23.1 for untransduced iNKT cells (online supplemental data 1B) with good transduction efficiency (57.7%±5.0%). Analyses of iNKT cells on day 0 and of untransduced iNKT cells and CD19-CAR-iNKT cells on day 20 revealed a preferential expansion of CD4+ cells and a decrease of the CD8+ population, while no consistent pattern could be identified for CD4−CD8− (DN) cells (online supplemental data 1C). Moreover, CD19-CAR-iNKT cells showed low activation (CD25−) and an effector memory (CD44+CD45RA−) phenotype (figure 1B). Importantly, flow cytometric analysis of expanded cells revealed that our protocol did not induce cell exhaustion, despite the prolonged culture time (figure 1C).

10.1136/jitc-2023-007829.supp1 Supplementary data

To assess their functional fitness, CD19-CAR-iNKT cells were challenged against SEM (CD19+ ALL cell line), Raji (Burkitt lymphoma cell line) and ALL patient blasts (about 95% blasts in PBMCs) at different ratios for 4 hours and analyzed by flow cytometry and ImageStream. Untransduced iNKT cells were used as negative control. Strikingly, CD19-CAR-iNKT cells showed a robust efficiency in eradicating tumor cells in comparison to untransduced iNKT cells. For instance, the calculated cytotoxic activity against SEM at a 5:1 ratio (E:T) achieved 6.8%±1.2% (untransduced iNKT cells) and 84.3%±8.6% (CD19-CAR-iNKT cells), while for Raji cells, it was 7.2%±4.1% (untransduced iNKT cells) and 65.8%±4.2% (CD19-CAR-iNKT cells). Patient blasts were very sensitive to CD19-CAR-iNKT cells, which showed 81.3%±3.3% of cytotoxic activity at a 5:1 ratio (6.7%±6.8% for untransduced iNKT cells) (figure 1D). Further, apoptosis induction of SEM by CD19-CAR-iNKT cells was also confirmed by ImageStream analysis (figure 1E).

Next, CD1d-mediated cytotoxicity of CD19-CAR-iNKT cells was evaluated by challenging them with CD19−CD1d+ Jurkat cells on treatment with α-GalCer or its vehicle DMSO. Interestingly, neither CD19-CAR-iNKT cells nor untransduced iNKT cells treated with DMSO reacted against the tumor cells, although a non-significant trend can be observed in favor of CD19-CAR-iNKT cells. Strikingly, the addition of α-GalCer induced a significant and robust cytotoxic activity for both untransduced and CD19-CAR-iNKT cells (figure 1F).

### CD19-CAR-iNKT cells use both CAR and CD1d pathways to induce death of target cells

**Figure 2:**
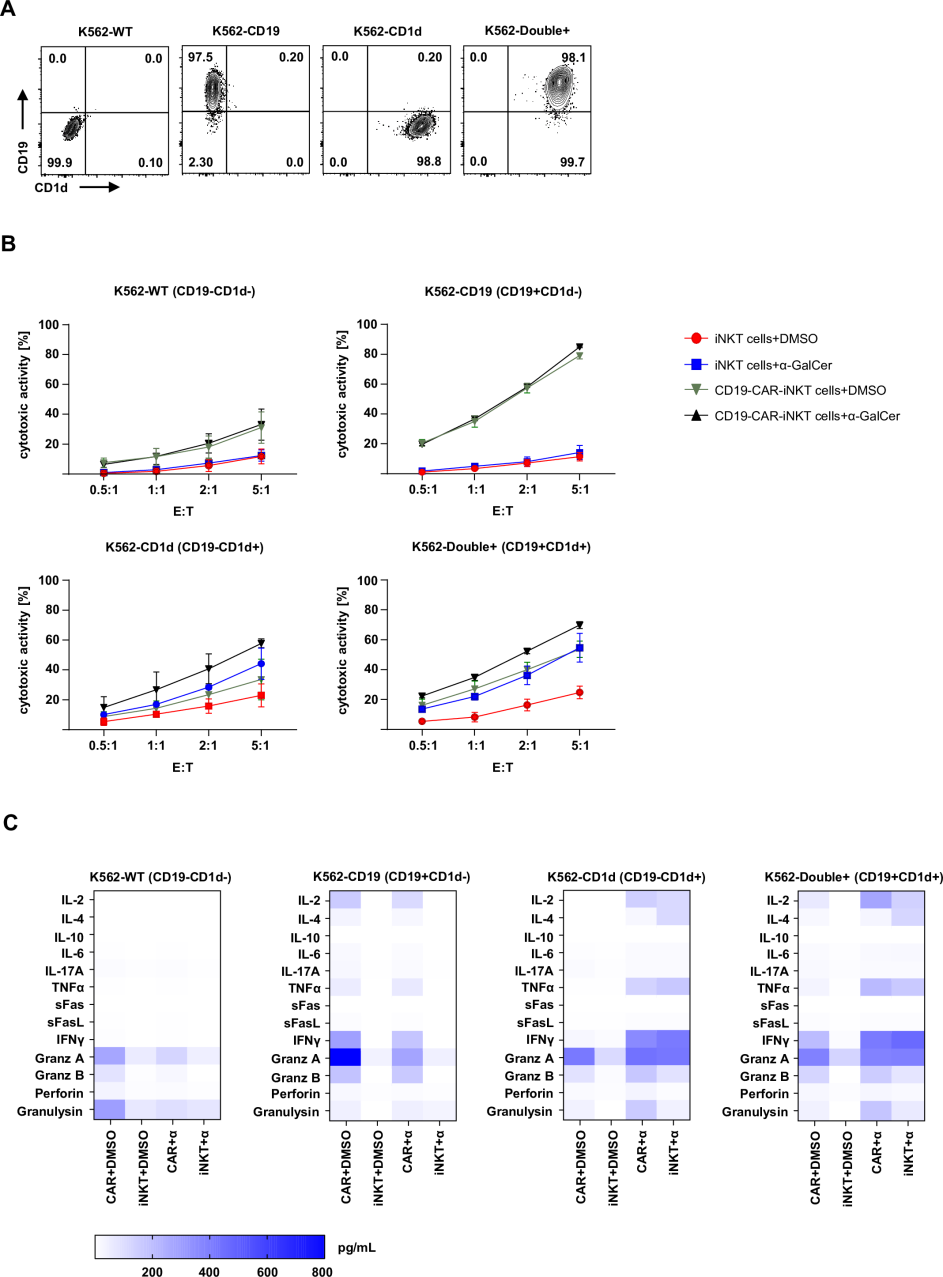
CD19-CAR-iNKT cells use both CAR and CD1d pathways to kill target cells. (A) Representative dot plots of CD19 and CD1d expression on modified K562. (B) Cytotoxicity of CD19-CAR-iNKT cells with and without α-GalCer against K562-WT, K562-CD19+CD1d−, K562-CD19−CD1d+ and K562-double+ (n=3). Data were obtained after gating on all cells (FSC-A vs SSC-A), singlets (SSC-A vs SSC-W) and annexin V−VD−CTV+ cells. (C) Heat maps showing pooled data of cytokine release by CD19-CAR-iNKT cells and untransduced iNKT cells on treatment with DMSO or α-GalCer and challenge with modified K562 cells after 4 hours (n=3). The results are expressed as pg/mL. The experiments were performed at least three times independently with three different iNKT-cell donors and in at least two technical replicates. Error bars show SEM. iNKT, invariant natural killer T.

Next, we evaluated which mechanisms and molecules are involved in the CD19 and/or CD1d-mediated cytotoxicity of CD19-CAR-iNKT cells. For that, CD19-CAR-iNKT cells were challenged for 4 hours with K562 cells modified to stably express CD19 and/or CD1d (figure 2A). Considering that α-GalCer seems to be necessary for CD1d-mediated cytotoxicity in vitro, the assays were performed in the presence of α-GalCer or DMSO. Cytotoxicity of CD19-CAR-iNKT cells against K562 wild-type (WT) cells was slightly enhanced in comparison to untransduced iNKT cells. However, no additional effect was observed on α-GalCer treatment. As expected, CD19-CAR-iNKT cells displayed strong cytotoxicity against K562-CD19 cells in a dose-dependent manner. α-GalCer did not promote any further enhancement of the cytotoxic activity in this setting. The cytotoxicity of CD19-CAR-iNKT cells was not higher than that of untransduced iNKT cells when tested against K562-CD1d. Here, α-GalCer treatment enhanced cytotoxicity in comparison with DMSO treatment. Finally, α-GalCer significantly enhanced the cytotoxicity of both untransduced iNKT cells and CD19-CAR-iNKT cells against K562-double+ cells (figure 2B).

These observations were also supported by the cytokine profile revealed through multiplex analysis of the supernatant from this assay (figure 2C). Interestingly, a slight release of granzyme A and granulysin was observed when CD19-CAR-iNKT cells were challenged with K562-WT cells, which could explain the ‘background’ cytotoxicity observed in this setting. An α-GalCer independent release of IL-2, TNF-α, IFN-γ and effector molecules could be observed by CD19-CAR-iNKT cells, but not by untransduced iNKT cells tested against K562-CD19. Further, assays with CD1d-expressing cells, confirmed the necessity of α-GalCer for cytokine release. A similar pattern as for CD19+ cells could be observed for double-positive target cells. α-GalCer seemed to boost TNF-α release by both effector cells. Thus, these observations show the potential of CD19-CAR-iNKT cells to eliminate CD19+ malignancies through their CAR, while promoting immunoregulation and tumor control through the engagement of the TCR:CD1d machinery.

### PD-1:PD-L1/PD-L2 interaction impairs the functionality of CD19-CAR-iNKT cells

**Figure 3:**
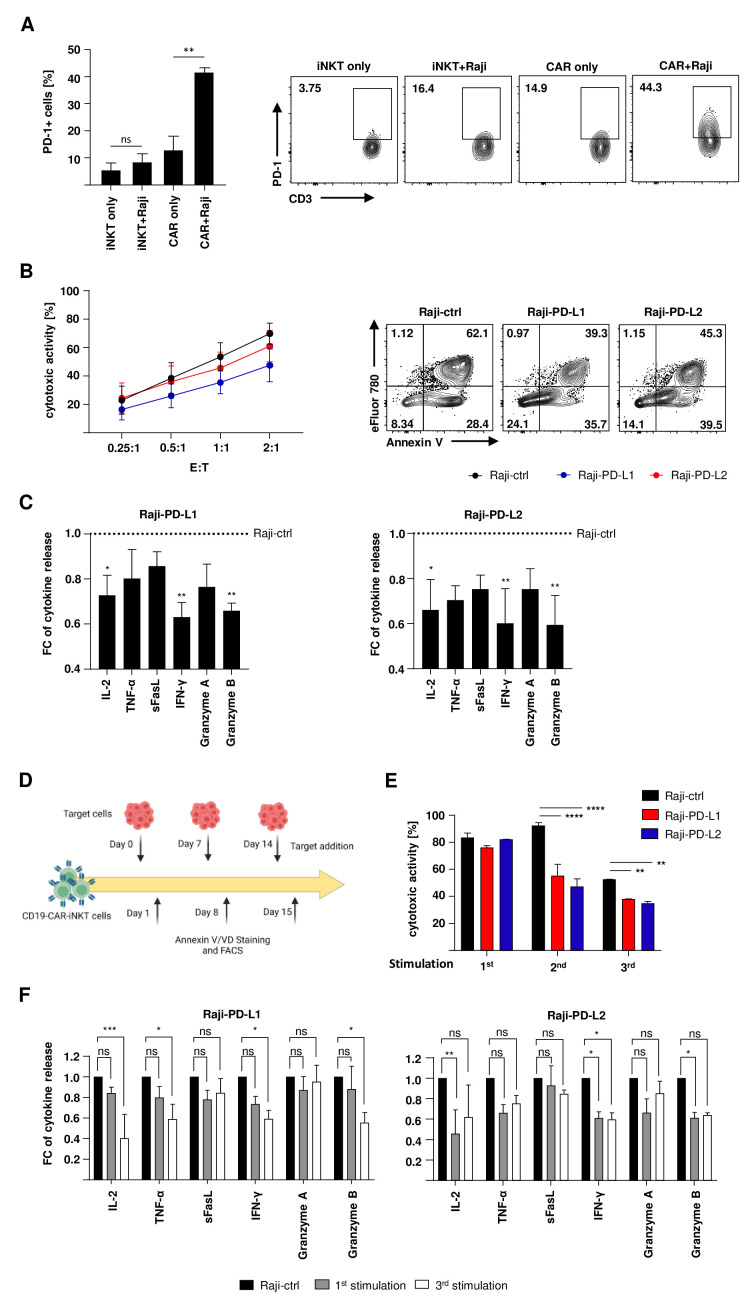
PD-1:PD-L1/PD-L2 interaction impairs the functionality of CD19-CAR-iNKT cells. (A) PD-1 expression on CD19-CAR-iNKT cells and untransduced iNKT cells on contact with Raji cells (left, n=3) and representative data for one donor (right). PD-1+ cells were analyzed from lymphocytes, singlets, alive iNKT cells or CD19-CAR-iNKT cells. (B) Cytotoxic activity of CD19-CAR-iNKT cells and untransduced iNKT cells against modified lymphoma cells for 24 hours (left, n=4) and representative dot plots for one CD19-CAR-iNKT-cell donor challenged with Raji-ctrl, Raji-PD-L1 and Raji-PD-L2. Annexin V/VD gates were set on singlets and CTV+ cells. (C) IL-2, TNF-α, sFasL, IFN-γ, granzyme A and granzyme B release by CD19-CAR-iNKT cells on contact with modified Raji-PD-L1 (left) and Raji-PD-L2 cells (right, n=3). The data are represented in fold-change (FC) of cytokine release normalized to the assay with Raji-ctrl as targets. (D) Scheme of the rechallenge assay. (E) Cytotoxicity of CD19-CAR-iNKT cells for one donor during three stimulation rounds with either Raji-ctrl, Raji-PD-L1 and Raji-PD-L2. Target cells were discriminated from CD19-CAR-iNKT cells through the FSC-A vs SSC-A parameters and CTV+ staining. Only singlets were analyzed. (F) Multiplex supernatant analysis after the first and third stimulation rounds of CD19-CAR-iNKT cells against Raji-PD-L1 (left) and Raji-PD-L2 (right) (n=3). The data are represented in fold-change (FC) of cytokine release normalized to the assay with Raji-ctrl as targets. The experiments were performed at least three times independently with at least three different iNKT-cell donors in two technical replicates. Error bars show SEM. Two-way ANOVA. ns p>0.05, *p≤0.05, **p≤0.01, ***p≤0.001. ANOVA, analysis of variance; iNKT, invariant natural killer T.

To test sustained cytotoxicity of CD19-CAR-iNKT cells, coincubation period with target cells was extended from 4 to 24 hours (online supplemental data 2A). Further analysis of CD19-CAR-iNKT cells revealed a significant increase in the frequency of PD-1+ cells on challenge with target cells (figure 3A) counteracting cellular activation and indicating exhaustion. Apart from exhaustion markers, functional assays are more adequate to better describe this phenomenon. Therefore, the functionality of CD19-CAR-iNKT cells was assessed while being challenged with Raji cells overexpressing PD-L1 (Raji-PD-L1) and PD-L2 (Raji-PD-L2). An empty vector was used as negative control (Raji-ctrl) (online supplemental data 2B). After 24 hours of coincubation, decreased cytotoxicity of CD19-CAR-iNKT cells against targets cells expressing PD-1 ligands was observed. This effect was most pronounced against PD-L1+ Raji cells (figure 3B). Interestingly, multiplex analyses of the supernatants from these assays also revealed a decreased release of several cytokines and effector molecules such IL-2, TNF-α, IFN-γ, granzyme A and B (figure 3C).

Next, CD19-CAR-iNKT cells were also tested in rechallenge assays to test long-term cytotoxicity. Therefore, part of the effector cells was restimulated with modified target cells every 7 days, while the other part was challenged for 24 hours with PD-L1/2+ cells (figure 3D). For the second and third stimulations, a more concise effect of the PD-L1/2 expression on cytotoxicity could be observed compared with the first round (figure 3E). Similarly, a reduction of the cytokine and effector molecule release could be noted, especially after the third stimulation round (figure 3F).

### Checkpoint inhibition increases cytokine release and cytotoxicity of CD19-CAR-iNKT cells

**Figure 4:**
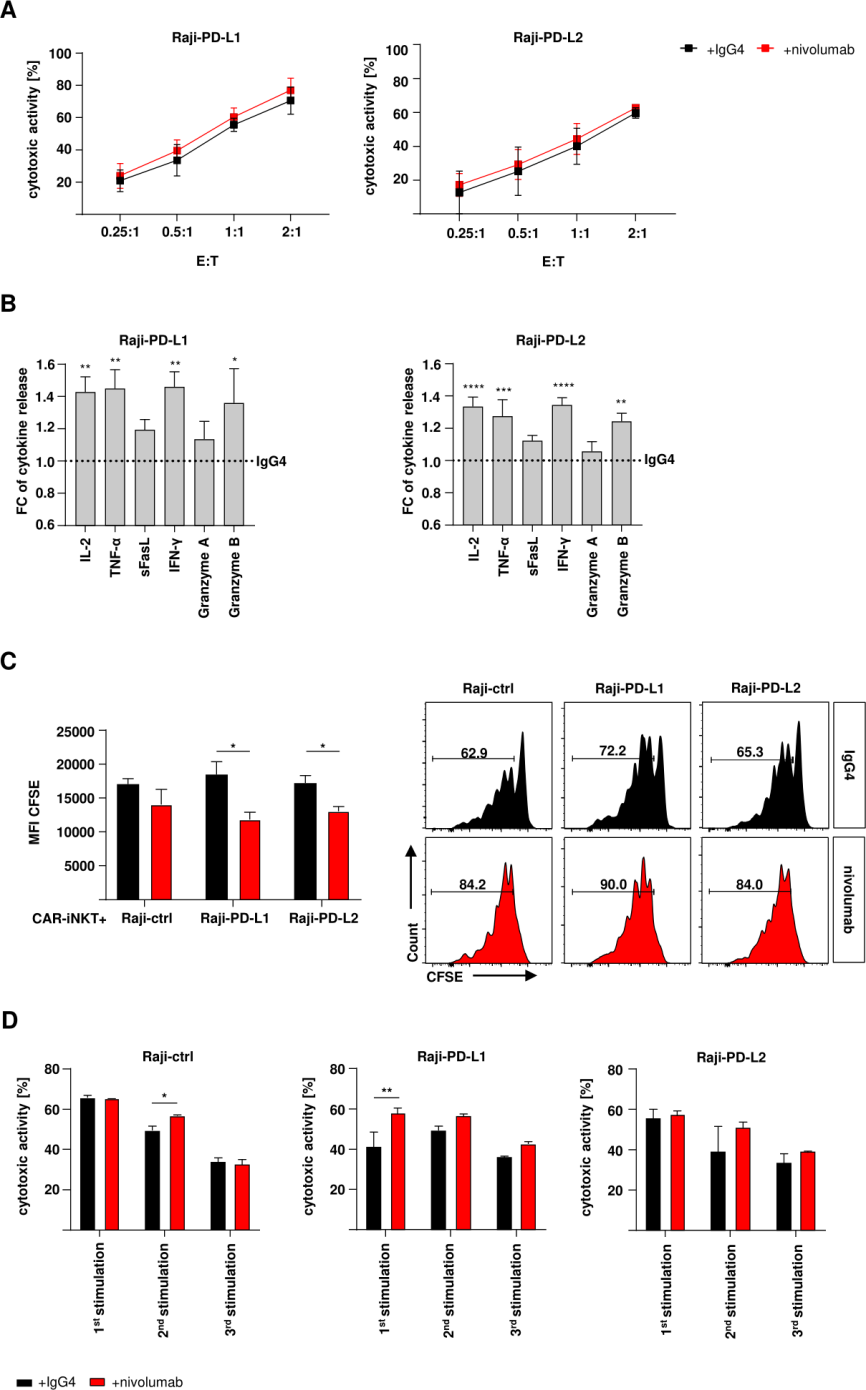
Checkpoint inhibition increases cytokine release by CD19-CAR-iNKT cells. (A) Cytotoxic activity of CD19-CAR-iNKT cells against modified lymphoma cells (n=4). Annexin V/VD gates were set on singlets and CTV+ cells. (B) IL-2, TNF-α, sFasL, IFN-γ, granzyme A and granzyme B release by CD19-CAR-iNKT cells on contact with modified Raji-PD-L1 (left) and Raji-PD-L2 cells (right) and under nivolumab/IgG4-treatment (n=5). The data are represented in fold change (FC) of cytokine release normalized to the assay with IgG4. (C) Proliferation of nivolumab (left) or IgG4-treated (right, n=3) CD19-CAR-iNKT cells on challenge with modified Raji cells and representative data for one donor. Gates were set on lymphocytes, singlets, live iNKT or CD19-CAR-iNKT cells. The results are expressed as mean fluorescence intensity (MFI). Error bars show SEM. (D) Representative data of the cytotoxic activity of CD19-CAR-iNKT cells against Raji-ctrl (left), Raji-PD-L1 (middle) and Raji-PD-L2 (right) with nivolumab or IgG4-treatment throughout three stimulation rounds (n=1). Error bars show SD. The experiments were performed at least three times independently with at least three different iNKT-cell donors in three technical replicates. Two-way ANOVA. ns p>0.05, *p≤0.05, **p≤0.01, ***p≤0.001, ****p≤0.0001. ANOVA, analysis of variance; iNK, invariant natural killer T.

Due to the effect of the PD-1:PD-L1/2 interaction, we used the PD-1 checkpoint inhibitor nivolumab to reverse the decreased functionality of CD19-CAR-iNKT cells. First, short-term cytotoxicity was assessed. Therefore, CD19-CAR-iNKT cells were challenged with PD-L1/2 expressing target cells for 24 hours with 10 µg/mL nivolumab or its IgG4 isotype control. Nivolumab did not significantly affect short-term cytotoxicity of CD19-CAR-iNKT cells against tumor cells expressing PD-1 ligands (figure 4A). Nevertheless, the release of some cytokines and effector molecules was improved from CD19-CAR-iNKT cells treated with nivolumab. While the release of sFasL and granzyme A was only slightly increased (less than 10%), an increase of approximately 50% could be observed for IL-2, TNF-α, IFN-γ and granzyme B (figure 4B). Further, the proliferation of CD19-CAR-iNKT cells was tested during checkpoint inhibition on antigen encounter. For that, CD19-CAR-iNKT cells were stained with CFSE and cocultured with PD-L1/2+ Raji cells for 5 days and analyzed by flow cytometry. CFSE dilution revealed that nivolumab significantly enhanced proliferation of CD19-CAR-iNKT cells in contrast to isotype control (figure 4C). Moreover, cytotoxic activity of CD19-CAR-iNKT cells was improved in rechallenge assays through treatment with nivolumab especially against PD-L1 expressing cells and after the second and third stimulation (figure 4D, online supplemental data 3A). Interestingly, multiplex analyses showed an overall improvement in the release of certain cytokines and effector molecules when nivolumab-treated CD19-CAR-iNKT cells were challenged with PD-L1 and PD-L2 expressing target cells. This effect was particularly pronounced for the release of granzyme B through cells challenged with Raji-PD-L1 (online supplemental data 3B). Importantly, when CD19-CAR-iNKT cells were tested against patient blasts on checkpoint inhibition, an overall higher cytokine release could be observed (online supplemental data 3C).

### CD19-CAR-iNKT cells maintain their ability to prevent alloreactive T-cell activation and proliferation

We have shown that CD19-CAR-iNKT cells efficiently lyse target cells. Their functionality can be further increased by checkpoint inhibition. Therefore, we wondered whether CD19-CAR-iNKT cells maintain their tolerogenic ability to prevent GVHD. We have previously shown that culture-expanded human iNKT cells prevent T-cell activation and proliferation through the preferential apoptosis of conventional DCs from transplanted patients in a CD1d-dependent manner.^8^ Here, we already showed that CD19-CAR-iNKT cells maintain their ability to promote CD1d-dependent cytotoxicity (figure 1F).

**Figure 5:**
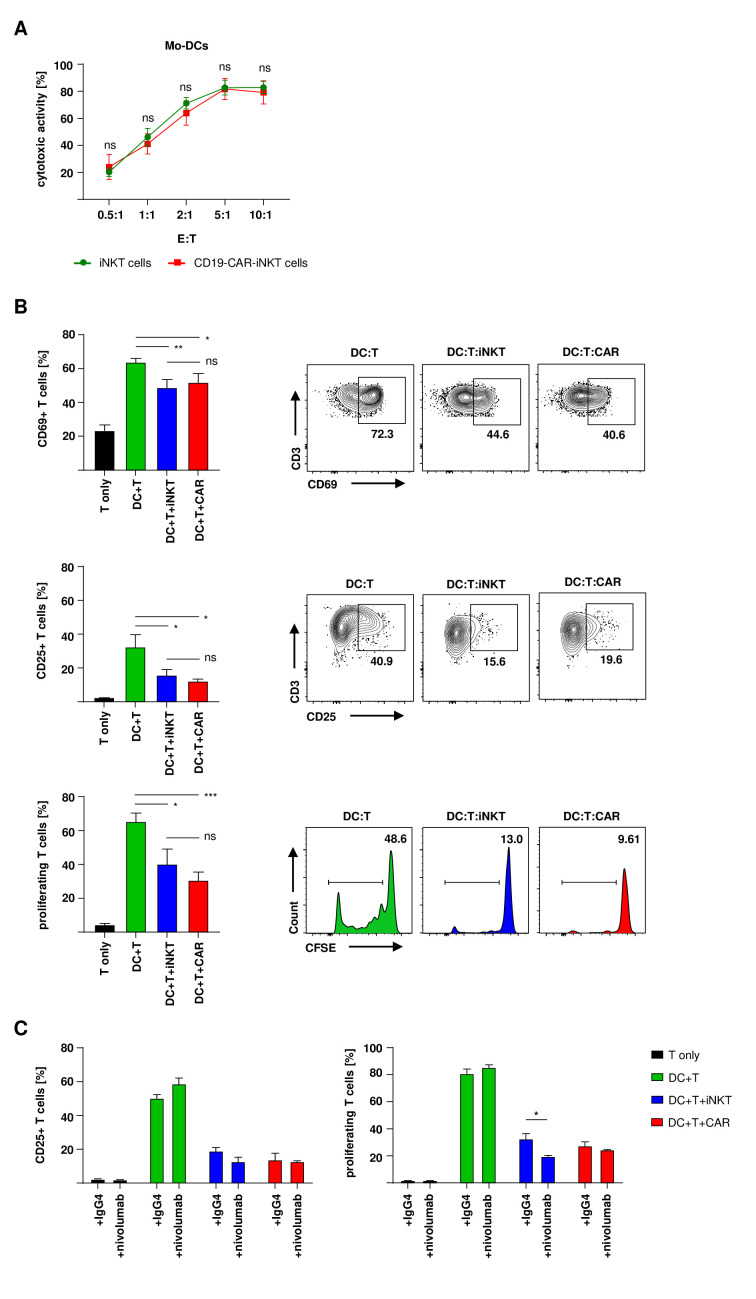
CD19-CAR-iNKT cells maintain their ability to prevent T-cell activation and proliferation. (A) Mo-DC-apoptosis induction by untransduced iNKT cells and CD19-CAR-iNKT cells (n=3). CD3+ iNKT cells/CD19-CAR-iNKT cells were excluded by gating on CD3− cells only. (B) Histograms and representative dot plots of early activation, late activation and proliferation of T cells (n=3). (C) T-cell activation on day three and proliferation on day seven after coincubation with mo-DCs in addition of either nivolumab or the isotype control (n=3). Cocultures without iNKT cells were used as positive control and T cells cultured alone were used as negative control. iNKT cells were excluded through gating on PBS57-CD1d-tetramer negative populations. Only live (VD−) T cells were analyzed. The experiments were performed at least three times independently with at least three different iNKT-cell donors in three technical replicates. Error bars show SEM. Two-way ANOVA, ns p>0.05, *p≤0.05, **p≤0.01, ***p≤0.001. ANOVA, analysis of variance; iNKT, invariant natural killer T; Mo-DC, monocyte-derived dendritic cell; PBS, phosphate-buffered saline.

First, CD19-CAR-iNKT cells and untransduced iNKT cells were tested against mo-DCs that were used as HLA mismatched stimulators of T cells in subsequent assays. After coculture, apoptosis of mo-DCs was analyzed by flow cytometry. Both CD19-CAR-iNKT cells and untransduced iNKT cells were able to induce DC apoptosis within 4 hours (figure 5A). After that, T-cell functionality was analyzed in an MLR. Therefore, MHC mismatched T cells and mo-DCs were cocultured for 7 days in presence of CD19-CAR-iNKT cells or untransduced iNKT cells. Cells were analyzed by flow cytometry on day 1 (for early activation), day 3 (late activation) and day 7 (proliferation). Importantly, both CD19-CAR-iNKT cells and untransduced iNKT cells maintained their ability to prevent T-cell activation (CD69 and CD25 expression) and proliferation (CFSE dilution) (figure 5B).

Next, we tested the effect of nivolumab on T-cell activation and proliferation in the presence of CD19-CAR-iNKT cells. First, the addition of nivolumab increased the activation and proliferation of alloreactive T cells in the absence of (CAR)-iNKT cells. Second, both untransduced and CD19-CAR-iNKT cells maintained or even improved their ability to decrease alloreactive T-cell activation and proliferation in the presence of nivolumab (figure 5C).

## Discussion

CARs are genetically engineered molecules designed to enhance the specificity and functionality of effector immune cells by recognizing antigens independent of their MHC restriction. A convincing body of evidence has demonstrated the efficacy and safety of CAR-bearing cells both in vitro and in vivo. The advent of second generation CARs led to FDA approval and a broader clinical application of this technology. Thus, CAR therapy has become a well-established therapeutic strategy, with high efficacy, especially against hematological malignancies. Nevertheless, application of CAR-T cells after allogeneic HCT remains challenging given the risk of severe GVHD. After allogeneic HCT, leukapheresis from the patient usually results in harvest of T cells from the stem cell donor.^33 34^ This risk of GVHD is related to MHC disparity, which is most pronounced in the haploidentical setting. However, the occurrence of GVHD is a major complication of allogeneic HCT, increasing therapy-related mortality per se. In addition, relapse is the most common cause of death after transplant.

In this sense, the idea of using CAR-iNKT cells is appealing since they are excellent killer cells and might prevent GVHD. Indeed, CAR-iNKT cells have already been investigated against various malignancies such as B-cell lymphoma, neuroblastoma and myeloma.^26–29^ Rotolo *et al* published a comprehensive study showing the potential of CAR-iNKT cells directed against CD19. In this work, CD19-CAR-iNKT cells showed enhanced functionality in a direct comparison to CD19-CAR-T cells both in vitro and in vivo.^26^ Here, the authors also found a cooperative activation of CD19-CAR-iNKT cells through CD1d and the invariant TCR. Furthermore, Simonetta *et al* assessed the direct antitumor effect of allogeneic CD19-CAR-iNKT cells against B-cell lymphoma in immunocompromised mice. CD19-CAR-iNKT cells exerted robust tumor control and elicited cross-priming of host CD8+T cells, enhancing long-term tumor control. This work underlines the duality of CD19-CAR-iNKT cells as both direct cytotoxic effectors but also as potent immunoregulators.^30^ Beyond, our present study shows high efficiency of CD19-CAR-iNKT cells containing CD137 and CD28 as costimulatory domains against cell lines from highly aggressive lymphoid malignancies and CD19+ patient blasts. Importantly, we have demonstrated that both CAR and CD1d pathways can be efficiently engaged. Since the immunoregulatory properties of iNKT cells relies on CD1d engagement, apoptosis induction of other immune cells and cytokine release, our data suggest that CD19-CAR-iNKT cells maintain and unveil their myriad of functionalities in an allogenic HCT setting. Also, Delfanti *et al* showed recently that TCR-expressing iNKT cells achieved tumor control by simultaneously modulating suppressive myeloid cells and actively killing malignant cells confirming the duality of genetically engineered iNKT cells.^35^ Therefore, genetically engineered iNKT cells combine special features making them ideal candidates for distinct clinical scenarios like allogeneic HCT.

Upregulation of inhibitory receptors such as PD-L1 and cadherin on tumor cells is a known mechanism of tumor evasion. The interaction with their counterparts on the surface of effector cells leads to the suppression of these cells and eventual loss of function and persistence.^36 37^ This effect has also been noted in CAR-T-cell therapy.^38 39^ In this context, the combination of CAR-T-cell therapy and checkpoint inhibition seems to be reasonable, with a few studies demonstrating its efficacy.^40–42^ However, this strategy has shown limited efficiency in patients who previously underwent allogeneic HCT probably due to insufficient cancer-directed immune responses. Moreover, an increased risk for GVHD has been observed, restricting its application in particular early after transplantation when the risk of relapse is most pronounced.^23 25^ Therefore, we focused on the effects of checkpoint inhibition on CD19-CAR-iNKT-cell functionality. Checkpoint inhibition is also a valuable tool to stress our MLR alloreactivity assays to test the ability of CAR-iNKT cells to prevent alloreactive immune responses. Checkpoint inhibitors applied together with iNKT cells has already been described and showed promising results.^43 44^ For instance, iNKT cells promoted tumor control with PD-1 checkpoint inhibition in a murine model of pancreatic cancer.^18^

However, the combination of CD19-CAR-iNKT cells and checkpoint inhibition has not been evaluated so far. In our study, nivolumab had only marginal effects on short-term cytotoxicity of CD19-CAR-iNKT cells against modified Raji cells. However, the production of several key cytokines and granzyme B was greatly enhanced. No improvement of short-term cytolytic function but enhancement of cytokine release by CD19-CAR-T cells was also observed in another recent study: Here, the CAR was designed to coexpress a PD-1/CD28 fusion protein, which converted the inhibitory effects of PD-1 engagement into positive signals.^45^ However, in the course of repetitive encounter assays, we found gradually improved cytotoxicity, which could be a result of increased cytokine release and enhanced proliferation of effector cells during each stimulation round. Another study using CAR-T cells demonstrated that PD-1 checkpoint inhibition through PD-1 blocking antibodies results in increased cytotoxicity, cytokine production and proliferation of CAR-T cells directed against mesothelioma.^39^ Likewise, proliferation assays revealed a significantly improved proliferation rate of effector cells treated with nivolumab. Wang *et al* also found that adding checkpoint inhibition to iNKT-cell stimulating glycolipids prevented tumor growth and resulted in sustained iNKT-cell numbers.^46^ Checkpoint inhibition promotes proliferation and survival of CAR-iNKT cells that are both critical for long-term tumor control.

Altogether, this work reinforces that CAR-iNKT cells are an attractive alternative to CAR-T cells to prevent or treat relapse after allogeneic HCT with the advantage of GVHD prevention and allowing immunostimulatory approaches such as checkpoint inhibition to further strengthen GVL effects.

## Data Availability

Data are available on reasonable request. All data relevant to the study are included in the article or uploaded as online supplemental information.
