# Supplementary material for: PD-1 checkpoint inhibition enhances the antilymphoma activity of CD19-CAR-iNKT cells that retain their ability to prevent alloreactivity
Source: J Immunother Cancer. 2024 Jan 31;12(1):e007829. doi: 10.1136/jitc-2023-007829 (PMC10831439; doi:10.1136/jitc-2023-007829)
Supplement: Supplementary data [file jitc-2023-007829supp001.pdf]

Supplementary data 1

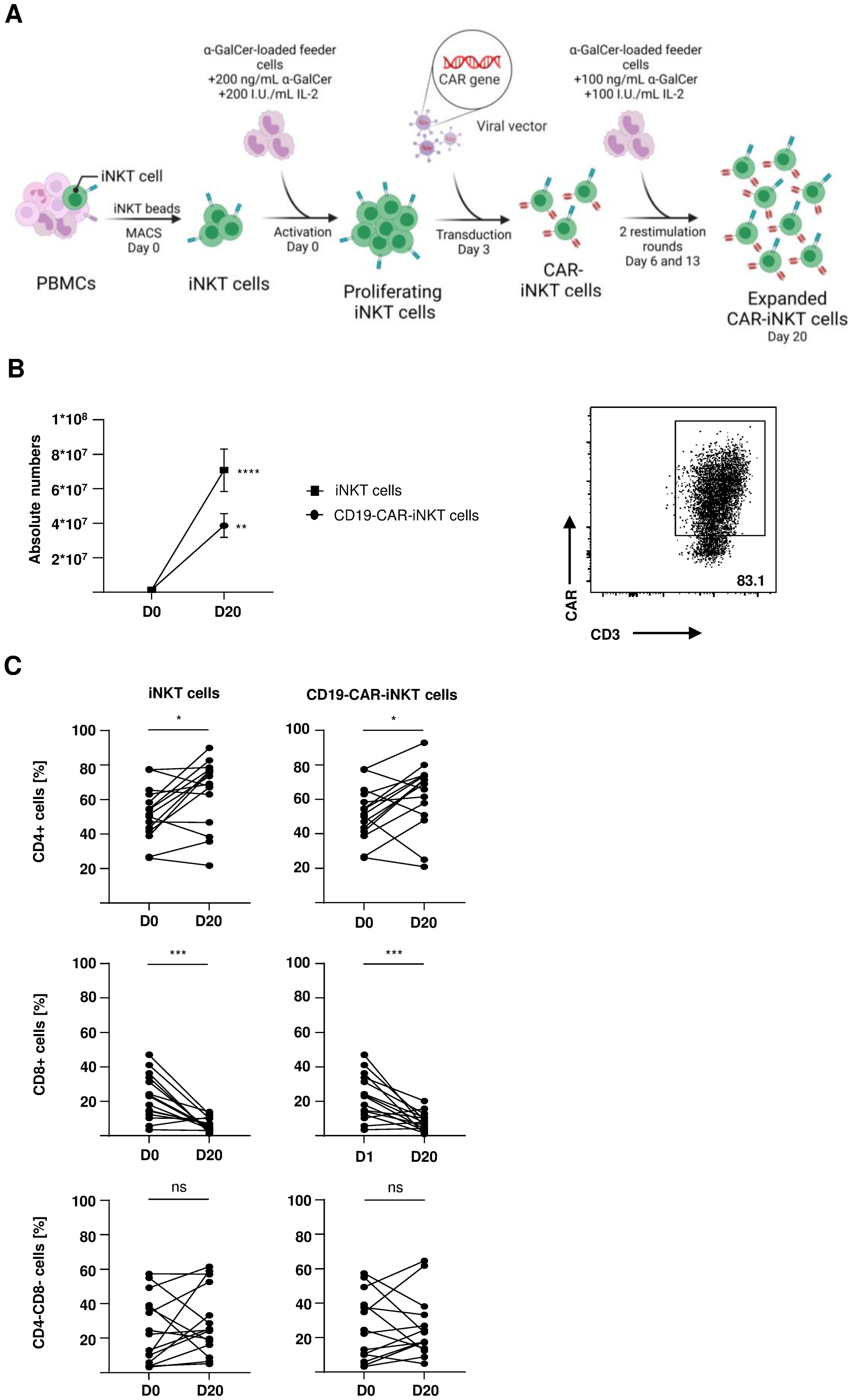

Supplementary data 1. CD19-directed CAR-iNKT cells induce cell death of CD19+ cell lines and primary patient blasts. (A) Scheme of the generation of CD19-CAR-iNKT cells. The figure was generated with Biorender® **(B)** Absolute numbers of expanded untransduced iNKT cells and CD19-CAR-iNKT cells on day 20 (left, n=10) and representative data showing the transduction efficiency for one donor (right). Expansion was determined by flow cytometry and cell counting. **(C)** Percentage of CD4, CD8 and double negative (CD4-CD8-) untransduced iNKT cells and CD19-CAR-iNKT cells on day 0 and day 20. Only live (VD-) iNKT cells/CD19-CAR-iNKT cells were included in the analysis. Error bars show SEM. Two-way ANOVA for **(B)** and paired *t*-test for **(C)**. ns p>0.05, * p≤0.05, ** p≤0.01, *** p≤0.001, **** p≤0.0001.

Supplementary data 2

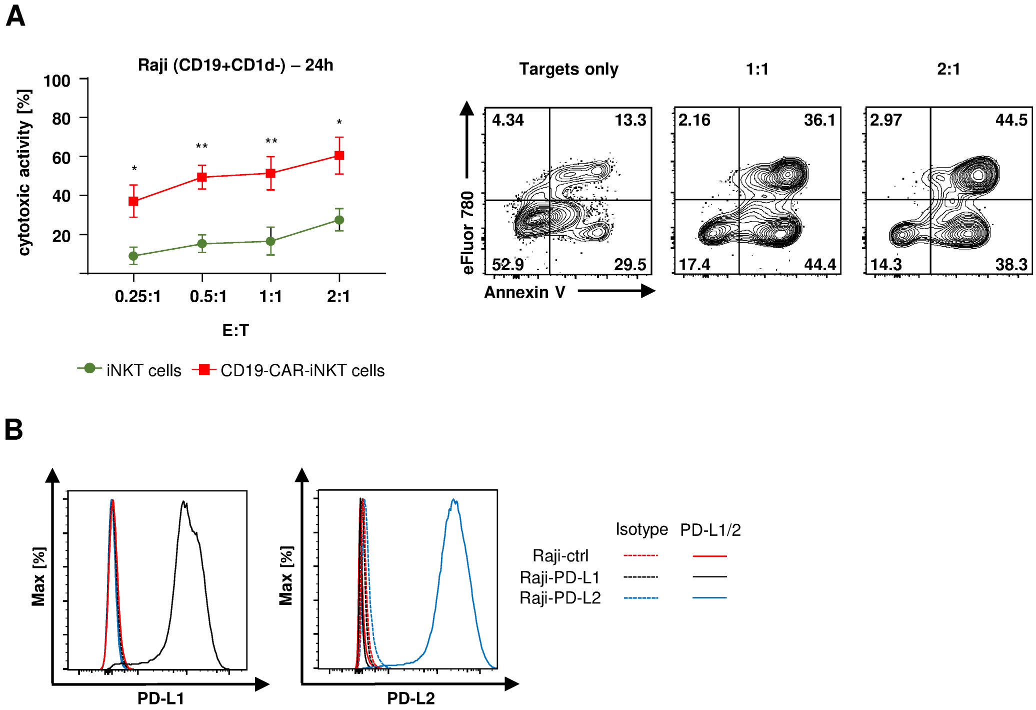

Supplementary data 2. PD-1:PD-L1/PD-L2 interaction impairs the functionality of CD19-CAR-iNKT cells. (A) Cytotoxic activity of untransduced iNKT cells and CD19-CAR-iNKT cells against Raji cells for 24h (left, n=3) and representative plots for targets only and target cells challenged with CD19-CAR-iNKT cells at 1:1 and 2:1 ratios (E:T) (right). Annexin V/VD gates were set on singlets and CTV+ cells. The experiments were performed at least three times independently with at least three different iNKT-cell donors in two technical replicates. **(B)** PD-L1 (left) and PD-L2 (right) expression by modified Raji cells. Stainings with isotype controls are shown as dashed lines, while specific staining is shown with full lines. Gates were set on singlets and live CD19+ cells. Error bars show SEM. Two-way ANOVA. * $p \le 0.05$, ** $p \le 0.01$.

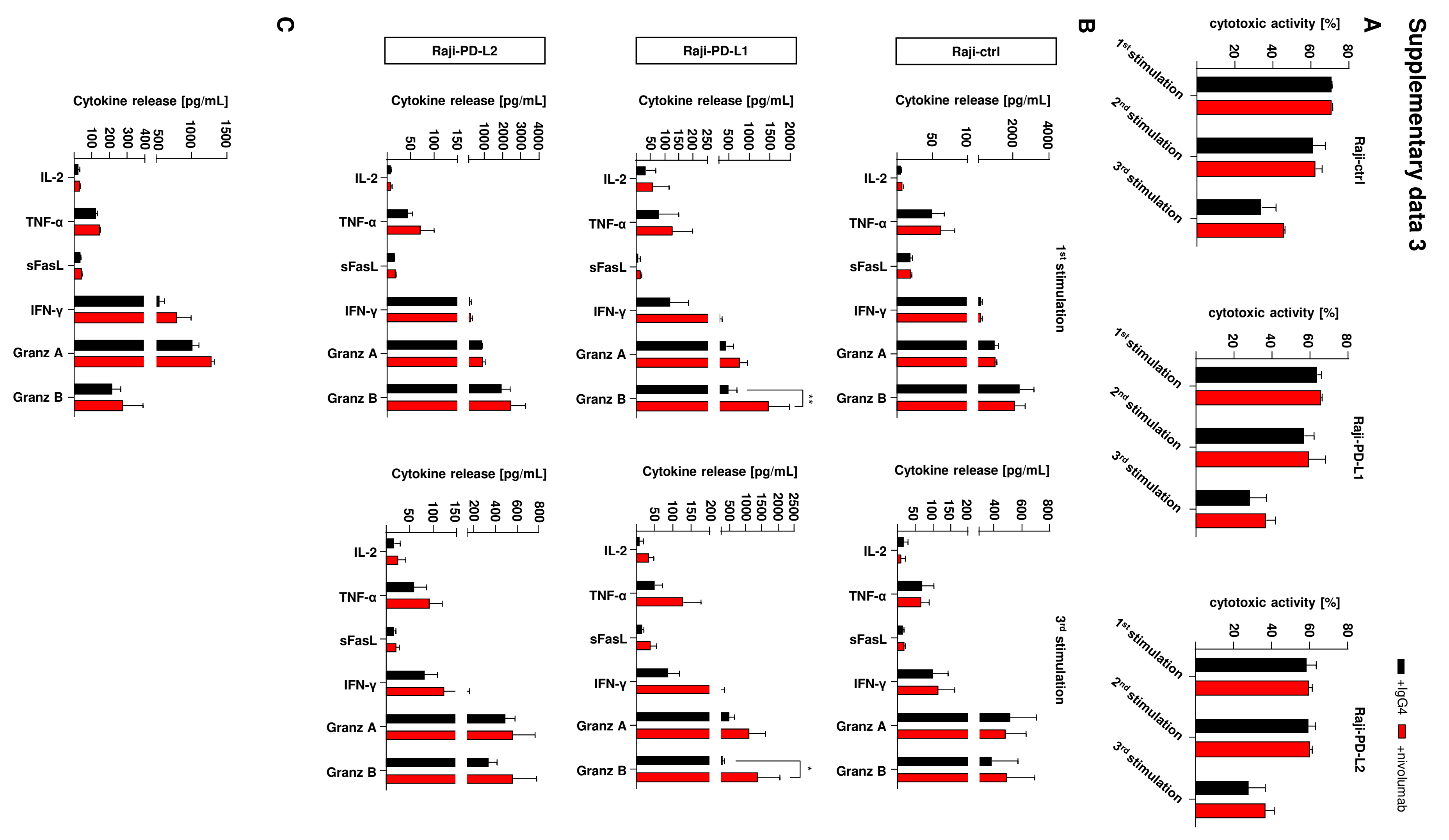
Supplementary data 3
A
Raji-ctrl
Raji-PD-L1
Raji-PD-L2
cytotoxic activity [%]
1st stimulation
2nd stimulation
3rd stimulation
+IgG4
+nivolumab
B
1st stimulation
3rd stimulation
Cytokine release [pg/mL]
IL-2
TNF-α
sFasL
IFN-γ
Granz A
Granz B
**
*
C

Supplementary data 3. Checkpoint inhibition increases cytokine release by CD19-CAR-iNKT cells. (A) Cytotoxicity of CD19-CAR-iNKT cells for one donor in the course of three stimulations with Raji-Ctrl (left), Raji-PD-L1 (middle) and Raji-PD-L2 (right). Target cells were discriminated from CD19-CAR-iNKT cells through the FSC-A vs. SSC-A gate and CTV+ staining. Only singlets were analyzed. Error bars show SD. **(B)** IL-2, TNF-α, sFasL, IFN-γ, granzyme A (Granz A) and granzyme B (Granz B) release of CD19-CAR-iNKT cells during 1st and 3rd stimulations with either Raji-ctrl, Raji-PD-L1 or Raji-PD-L2 in presence of IgG4 or nivolumab (n=3). Error bars show SEM. Two-way ANOVA. * $p \leq 0.05$, ** $p \leq 0.01$. Non-significant statistical analysis ($p > 0.05$) is not depicted. **(C)** Representative cytokine release (IL-2, TNF-α, sFasL, IFN-γ, granz A and granz B from CD19-CAR-iNKT cells upon challenge with patient blasts in the presence of IgG4 or nivolumab after 24h of co-incubation (n=1). Error bars show SD. The experiments were performed at least three times independently with at least three different iNKT-cell donors in three technical replicates.
